# PETRA IV: the ultralow-emittance source project at DESY

**DOI:** 10.1107/S1600577518008858

**Published:** 2018-08-09

**Authors:** Christian G. Schroer, Ilya Agapov, Werner Brefeld, Reinhard Brinkmann, Yong-Chul Chae, Hung-Chun Chao, Mikael Eriksson, Joachim Keil, Xavier Nuel Gavaldà, Ralf Röhlsberger, Oliver H. Seeck, Michael Sprung, Markus Tischer, Rainer Wanzenberg, Edgar Weckert

**Affiliations:** a Deutsches Elektronen-Synchrotron (DESY), Notkestrasse 85, 22607 Hamburg, Germany; b Department Physik, Universität Hamburg, Luruper Chaussee 149, 22761 Hamburg, Germany; c MAX IV Laboratory, Lund University, PO Box 118, SE-221 00 Lund, Sweden

**Keywords:** PETRA IV, ultralow-emittance source, diffraction-limited storage ring

## Abstract

The PETRA IV project aims at upgrading the present synchrotron radiation source PETRA III at DESY into an ultralow-emittance source. Being diffraction limited up to X-rays of about 10 keV, PETRA IV will be ideal for three-dimensional X-ray microscopy of biological, chemical and physical processes under realistic conditions at length scales from atomic dimensions to millimetres and time scales down to the sub-nanosecond regime. In this way, it will enable groundbreaking studies in many fields of science and industry, such as health, energy, earth and environment, mobility and information technology. The science case is reviewed and the current state of the conceptual design is summarized, discussing a reference lattice, a hybrid multi-bend achromat with an interleaved sextupole configuration based on the ESRF-EBS design, in more detail as well as alternative lattice concepts.

## Abstract 2

The PETRA IV project aims at upgrading PETRA III into an ultralow-emittance synchrotron radiation source.

## 1. Introduction   

**Figure 1:**
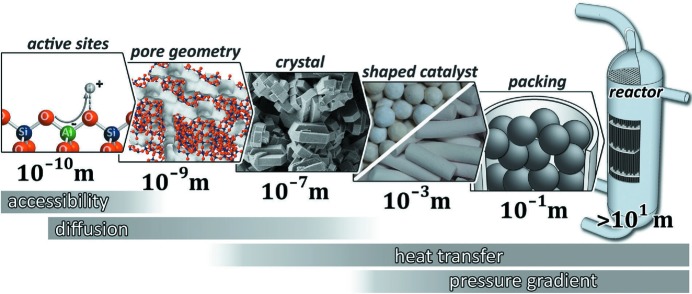
In order to understand a catalytic reaction inside a chemical reactor, the chemical and physical processes involved need to be followed under working conditions and on a broad range of length scales, from the atomic range to the dimensions of the reactor. Reproduced from Schwieger *et al.* (2016 ▸) with permission of The Royal Society of Chemistry.

Modern society strongly depends on the development of new solutions to the grand challenges in the fields of health, energy, earth and environment, mobility and information technology. They require a deep understanding of the complex physical, chemical and biological processes in nature. Synchrotron radiation sources are significantly contributing to understanding these processes, *e.g.* by determining complex structures in matter and their temporal evolution over a wide range of length and time scales. For a complete investigation of complex materials and processes on all relevant length scales [*cf.* Fig. 1 ▸ (Schwieger *et al.*, 2016 ▸)], however, today’s synchrotron radiation sources are limited in spectral brightness.

**Figure 2:**
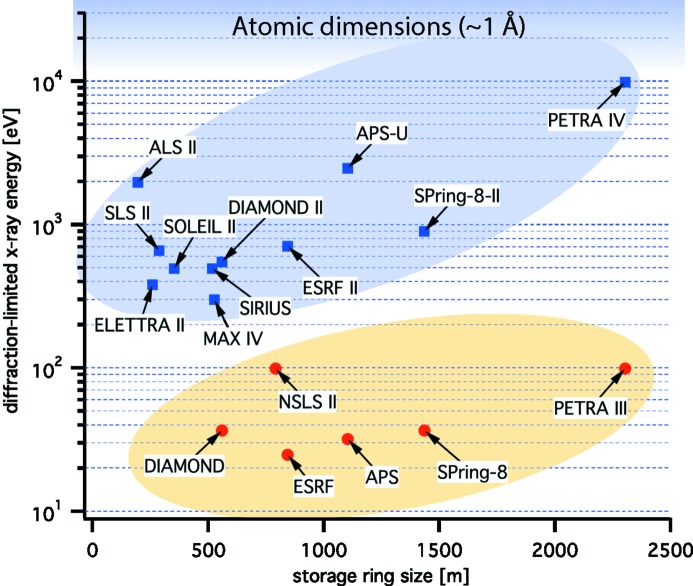
Diffraction limit for some synchrotron radiation sources and their future upgrades. The diffraction-limited X-ray energies were calculated based on emittances from Table 1 in Weckert (2015 ▸).

In recent years, a new (fourth) generation of light sources has been emerging based on new storage-ring lattice types, *i.e.* the multi-bend achromat (MBA) in several variants (Einfeld *et al.*, 1994 ▸, 2014 ▸; MAX-Lab, 2010 ▸; ESRF, 2014 ▸). The new concept allows for a significant reduction of the horizontal emittance compared with existing facilities. The synchrotron radiation source MAX IV in Lund, Sweden, is the first light source to be successfully commissioned with this new lattice type (Eriksson *et al.*, 2016 ▸), and SIRIUS in Campinas, Brazil, will be the next source based on this technology and is currently under construction. Starting in winter 2018, the European Synchrotron Radiation Facility (ESRF) in Grenoble, France, will undergo an upgrade to the fourth-generation light source ESRF-EBS with an emittance as low as 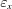 ≃ 130 pm rad at a beam energy of 6 GeV (Liuzzo *et al.*, 2016*a* ▸). Many other sources worldwide have upgrade plans along these lines, among which are also the high-energy storage-ring sources Advanced Photon Source (APS) at Argonne National Laboratory near Chicago, USA, aiming for an emittance of 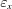 = 41–67 pm rad (Borland, 2014 ▸), and SPring-8 in Harima, Japan. The new technique allows for increased spectral brightness by one to two orders of magnitude and will dramatically change the landscape of synchrotron radiation facilities in the next decade (*cf.* Fig. 2 ▸).

As the emittance scales favourably with storage ring size and as PETRA has a particularly large circumference (2304 m), it offers the unique opportunity to push the generation of synchrotron radiation to its physical limits. DESY plans a major upgrade of PETRA III to the ultralow-emittance source PETRA IV. With a horizontal emittance in the range 10–30 pm rad and a vertical emittance smaller than 10 pm rad, PETRA IV would reach the diffraction limit for X-rays of up to 10 keV in both directions (*cf.* Fig. 2 ▸). In this way, PETRA IV would become the ultimate X-ray microscope, enabling users to follow chemical and physical processes in complex materials *in situ* and on length and time scales from atomic dimensions to millimetres and from nanoseconds to hours, respectively.

The PETRA IV project was initiated in spring 2016 and is currently in its conceptual design phase. This comprises a detailed analysis of the scientific case, driven by the requirements of the various science communities and new experimental opportunities, a conceptual design of the accelerator, storage ring facilities and new photon science beamlines. The completion of the conceptual design report (CDR) is planned for the beginning of 2019. The CDR will be the basis of the next project phase (technical design) of PETRA IV until 2021. It is planned that the PETRA IV construction phase will start with the pre-production of components and the preparation of the logistics. In the middle of the next decade, PETRA III shall be shut down, and PETRA IV shall go into operation after a shutdown period of about two years.

## 2. Science case   

The fundamental processes that determine everyday life, ranging from biological processes in living cells and chemical reactions inside a catalytic reactor to phase transformations in materials and friction in engines, encompass an enormous range of length scales, extending from atomic distances to macroscopic dimensions. The grand challenges to solving key global problems in the fields of health, energy, mobility and information technology are intimately related to a deep understanding of these processes and the underlying fundamental interactions under realistic processing or working conditions. Missing links, which connect properties on the nanoscale with those observed on macroscopic dimensions, prevent essential breakthroughs in these fields. The scale-bridging imaging of structures and their dynamics under *in vivo*/*in situ* and working conditions is therefore indispensable to unravel emergent functionalities in nature.

True progress in these fields will only be possible if all relevant length scales can be explored simultaneously. At PETRA IV, X-ray analytical techniques, such as X-ray fluorescence, absorption, diffraction, elastic, inelastic, and magnetic scattering, nuclear resonance and X-ray correlation spectroscopy, shall be combined with X-ray microscopy, giving access to element distributions, chemical states, local atomic structure, and electronic, vibrational and magnetic properties on all relevant length scales (*cf*. §2.2).

### 2.1. Science and industrial drivers   

PETRA IV can make significant contributions to many fields of science. In the following, a few non-exclusive examples are given in various different areas.

#### 2.1.1. Health   

The high brightness of PETRA IV will enable the routine structure determination of proteins and enzymes, from which only very small crystals can be grown and that are not accessible to conventional protein crystallography. This is the case for the large class of membrane proteins, which are responsible for the communication of the cell with its environment and thus play an eminently important role in infection research and as drug targets. The high brightness allows for a better definition of the beam, matching the beam size to the crystal and reducing the background scattering without loss in reciprocal-space resolution and flux. Single-pulse exposures in pump–probe schemes (Beyerlein *et al.*, 2017 ▸) will elucidate the relationship between structure and function of biological systems, and fine-focused X-ray imaging of cell organelles will provide access to pathogen pathways to develop new drugs against infection.

#### 2.1.2. Energy research   

PETRA IV will provide detailed insights into the atomic and molecular basis of energy generation in novel solar cells to improve their properties and efficiency. In new materials for batteries for sustainable and efficient energy storage, it will be possible to visualize the relevant chemical and ageing processes from macroscopic dimensions down to the atomic level. Microscopic studies of the chemistry of low-*Z* elements in the bulk, such as Li in a battery, will become feasible by combining inelastic X-ray scattering (IXS) with scanning microscopy (*cf*. §2.2.1).

#### 2.1.3. Mobility   

The combination of focused X-rays with their high penetration capability allows insight into processes of friction and abrasion in motors and generators, opening up the possibility to understand and control the fundamental processes on the way to avoid friction losses and reduce wear. Another field of application is the mapping of chemical processes from elementary atomic steps to macroscopic dimensions, *e.g.* to reduce emissions. The high degree of coherence of the radiation from PETRA IV will make it possible to monitor chemical reactions in a catalytic reactor under operating conditions at all relevant length scales. Studies in this area will help to find new highly efficient catalytic processes to further reduce unwanted emissions.

#### 2.1.4. Materials for information technology   

The ever-increasing demands on integration density and processing speed of information requires knowledge of the structure, chemical composition, magnetic order and electronic properties of new materials under *operando* conditions, from the atomic scale to the dimensions of entire circuits. PETRA IV will be able to map relevant processes inside these materials and devices. An important example is the investigation and optimization of fast switching processes in individual magnetic nanostructures, which serve as the smallest storage unit of information.

#### 2.1.5. Synthesis of complex materials   

With PETRA IV, chemical reactions inside a catalytic reactor can be followed under working conditions (Grunwaldt & Schroer, 2010 ▸; Schwieger *et al.*, 2016 ▸) (*cf*. Fig. 1 ▸) to improve catalysts in view of activity, selectivity and ageing, and push the efficiency, cost and sustainability of (industrial) processes to synthesize products such as fuels, fertilisers and health products. Under conditions of extreme pressure and temperature in high-pressure cells, it is possible to synthesize materials with exceptional properties that would be unthinkable under normal conditions. With PETRA IV, the structure formation under extreme conditions can be tracked along with the development of electronic and magnetic properties in minute sample volumes of less than 1 µm^3^. These research opportunities are essential for the elucidation of ordering processes and the formation of electronic correlations in materials, *e.g.* high-*T*_c_ superconductors. The development of highly stable composites will also greatly benefit from PETRA IV’s ability to correlate macroscopic properties under working conditions with properties at all other scales down to atomic interactions, *e.g.* the three-dimensional structure of micro- and nano-structured materials and alloys, in order to develop new tough and highly stable compound materials.

#### 2.1.6. Earth and environment   

PETRA IV will enable monitoring of the uptake and transport of chemicals and heavy metals in samples from the lithosphere and biosphere with highest sensitivity and spatial resolution in the nanometre range. This makes it possible to study their role in the formation of minerals, rocks and soils, for example to elucidate pollution pathways within the biosphere. Furthermore, specially designed diamond high-pressure cells will provide means to simulate the extreme pressure and temperature conditions inside the Earth and other planets, which is possible only in smallest volumes of a few µm^3^. Such small sample sizes can only be investigated with extremely intense, small and background-free X-ray beams. The nanofocused high-brightness beams of PETRA IV will thus contribute to answering questions on planet formation and other astrophysical processes.

The main commonality of most examples above is following physical and chemical processes under realistic conditions with the appropriate spatial and temporal resolution. Various X-ray analytical contrasts (*cf*. §2.2.1) give access to different physical and chemical properties. The significantly increased spectral brightness of PETRA IV improves the spatial and/or temporal resolution, improves the sensitivity and reduces background.

### 2.2. Methods and instrumentation enabling new science at PETRA IV   

Most synchrotron-radiation-based techniques at PETRA IV will strongly benefit from the increased spectral brightness, small source size and divergence. In the following, the most important methodological and instrumental science drivers are highlighted, in particular in view of the upcoming unique science opportunities.

#### 2.2.1. Bridging all spatial length scales: X-ray microscopy with coherent light   

The key to simultaneously covering all characteristic length scales of a complex system is the preparation of X-rays in a well defined state, *i.e.* in a phase space volume that comprises as few states as possible. In that case, (aberration free) X-ray optics can focus the beam to Abbe’s diffraction limit, *i.e.*

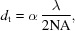
where λ is the X-ray wavelength, α is a constant close to unity given by the aperture function of the optics and NA is its numerical aperture. All length scales 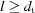 can be accessed directly in real space by imaging or scanning microscopy. At the same time the speckle size (smallest resolvable unit) in reciprocal space is proportional to 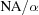. Thus all lateral length scales
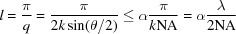
can be accessed in reciprocal space. Here, *q* is the momentum transfer of the scattered light, *k* its wavenumber and θ the full scattering angle. An example of a technique taking advantage of this is ptychography (Rodenburg & Faulkner, 2004 ▸; Thibault *et al.*, 2008 ▸; Dierolf *et al.*, 2010 ▸), also known as scanning coherent diffraction microscopy. It exploits the coherence to image samples with spatial resolution well beyond the beam size of the X-rays (Schropp *et al.*, 2012 ▸). Today, this technique is limited by the coherent flux,
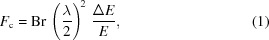
where Br is the spectral brightness and 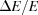 is the spectral bandwidth. For the presently brightest X-ray sources, such as PETRA III at DESY, the coherent flux is limited by its horizontal emittance and reaches about 0.1–1% of the total monochromatic radiation emitted by an undulator in the hard X-ray range (10 keV). Currently, this limits these techniques to studies with spatial resolutions down to the 10 nm range, leaving a *mesoscopic gap* of length scales that is not accessible today.

With emittances 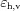 (both horizontal and vertical) in the region of 10 pm rad, X-ray beams will be diffraction-limited at about 10 keV, *i.e.*

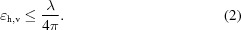
This implies that (nearly) the full beam of an undulator is laterally coherent, *i.e.* the lateral coherence length is in the range of the beam size, and can be focused to the diffraction limit (Schroer & Falkenberg, 2014 ▸). With this and with some improvements in X-ray optics (see, for example, Bajt *et al.*, 2017 ▸), the *mesoscopic gap* can be closed, giving access to all length scales down to the atomic level for coherent imaging techniques. To reach down in resolution to the atomic level, significant efforts will be needed to match the mechanical stability of the instrumentation. Alternatively, the increased coherent flux can be used to follow faster changes in the sample at a resolution of a few nanometre and above or to scan larger sample volumes.

Diffraction-limited focusing of the full undulator beam will have significant impact on all X-ray analytical techniques, such as X-ray fluorescence (XRF), scattering and diffraction (XRD) both in the small-angle (SAXS) and wide-angle (WAXS) regime, absorption spectroscopy (XAS), inelastic X-ray scattering (IXS), nuclear resonant scattering (NRS), X-ray beam-induced current (XBIC) and correlation spectroscopies [photon and fluorescence correlation spectroscopies (XPCS, XFCS), including cross-correlation analysis (XCCA)]. All these techniques can then be used efficiently as contrast mechanisms in scanning microscopy. So far, photon-hungry techniques, such as IXS and NRS, have not been able to be combined with high-resolution microscopy. With a diffraction-limited source in the hard X-ray range, they will be made available for nanoscopic investigations, giving, for example, access to local chemical information on low-*Z* elements in the bulk (*e.g.* the chemistry of Li inside a battery) (Huotari *et al.*, 2011 ▸). In addition, local electronic and magnetic properties that often vary on the nanometre scale (Lang *et al.*, 2002 ▸; Griffin *et al.*, 2012 ▸) will become accessible with PETRA IV.

#### 2.2.2. Bridging time scales: X-ray correlation spectroscopies   

**Figure 3:**
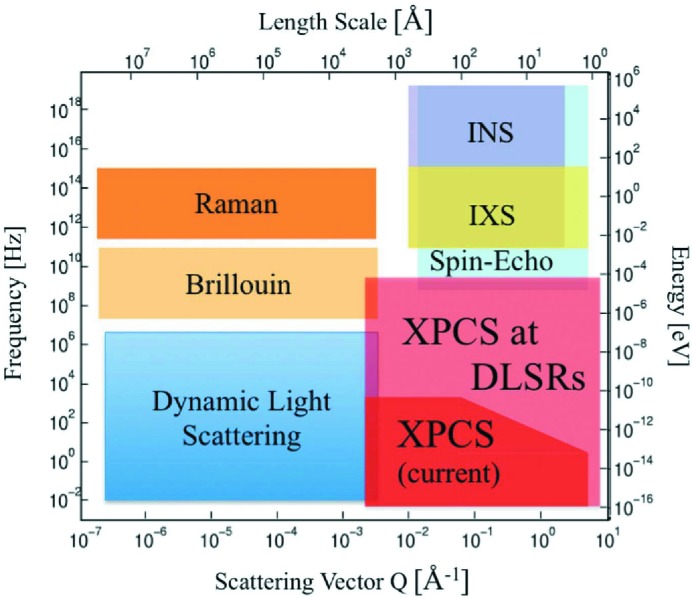
Map of spectroscopic techniques and their range in space and time. Currently, XPCS covers time scales down to the millisecond regime. At a diffraction-limited storage ring (DLSR) source, the accessible time range is extended down to the nanosecond regime. Reproduced from Shpyrko (2014) ▸ with permission of IUCr.

X-ray photon correlation spectroscopy (XPCS) is a powerful tool for studying dynamics in complex disordered systems (Grübel & Zontone, 2004 ▸; Shpyrko, 2014 ▸). Today, XPCS studies show dynamics (from milliseconds to minutes) on nanometre length scales in complex materials (Fig. 3 ▸). While other spectroscopic techniques and X-ray free-electron laser sources cover time scales from femtoseconds up to several nanoseconds at these length scales, there is a *temporal gap* ranging from several nano­seconds to milliseconds that is currently not accessible by any experimental technique. Covering this range, however, is crucial to fully understand processes in complex matter, for example diffusion processes in aqueous solutions.

The accessible time scales are limited by the signal-to-noise ratio 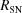 of correlation functions retrieved from a series of coherent scattering patterns, given by (Falus *et al.*, 2006 ▸; Shpyrko, 2014 ▸)
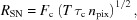
where 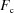 is the coherent flux [*cf*. equation (1)], *T* is the length of the data acquisition, 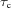 is the characteristic time scale to be studied in the system, and 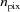 is the number of detector pixels. This means, that, for the same 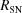, an increase of coherent flux by a factor *K* can be traded into a decrease of 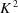 of the quantities under the square-root term. For example, for a given 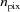 and data acquisition time *T*, an increase in coherent flux by a factor of 100 allows the investigation of the dynamical processes with a 10000-fold shorter characteristic time 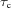. Similarly, for a given system with characteristic time 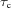, the acquisition time can be 10000-fold reduced, allowing the study of non-stationary and transient dynamics.

PETRA IV will thus enable several types of studies that are not possible with current sources:

(i) Follow systems with fast dynamics down to nanoseconds. This will, for example, enable the study of diffusion dynamics in aqueous solutions (Perakis *et al.*, 2017 ▸), *e.g.* in biology and chemistry.

(ii) Study non-equilibrium systems and transient behaviour, such as sliding or avalanche dynamics.

(iii) Combine with efficient nanofocusing, image dynamic spatial heterogeneity, for example in polymers, or study the dynamics of individual nano-objects.

Thus, PETRA IV closes the *temporal gap for dynamical processes at nanometre length scales*, giving access to the sub-microsecond range by X-ray correlation spectroscopy.

#### 2.2.3. High-energy X-rays: *in situ* and *operando* studies and bulk structure   

**Figure 4:**
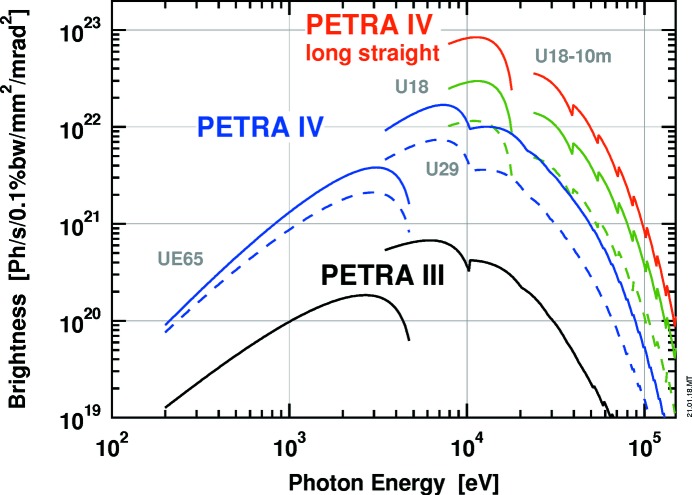
Spectral brightness for PETRA IV (blue, green and red curves) in comparison with PETRA III (black curves) for a ring current of 100 mA. For the soft X-ray spectrum, the brightness is compared for a 5 m UE65 undulator that is currently installed at beamline P04 at PETRA III. In the hard X-ray range, the comparison is made for a 5 m U29 undulator installed at P10. The full lines correspond to the high-brightness/high-coherence mode, and the dashed lines to the timing mode. In the high-energy X-ray range, significant gains in brightness can be achieved with a U18 undulator, *i.e.* a 5 m device in a regular ID section (green curve) and a 10 m device in one of the four long straight sections with optimized electron beam parameters.

As a high-energy storage ring with an electron energy of 6 GeV, PETRA IV will provide both high brightness and high flux for high X-ray energies above 30 keV. For high-energy X-rays the beam will not be diffraction limited [*cf*. equation (2)]. However, the reduction in emittance will translate nearly one-to-one into an increase in spectral brightness (*cf*. Fig. 4 ▸ for a comparison with PETRA III). This opens unparalleled possibilities for high-energy X-ray techniques, in particular for *in situ* and *operando* experiments that require complex sample environments, *e.g.* in solid-state physics, chemistry and biology, geoscience, nanotechnology and engineering materials science.

With the high brightness comes the possibility to focus the high-energy X-rays far more efficiently than today, fostering spatially resolved experiments at high photon flux. This enables high-energy X-ray microscopy with various contrasts, such as diffraction and scattering for the determination of the hierarchical structure of engineering materials (Simons *et al.*, 2014 ▸), and pair distribution function (PDF) ana­ly­sis. With the latter technique, the local structure can be determined also for non-crystalline and amorphous materials. The combination with tomo­graphy will allow high-resolution three-dimensional maps of the local atomic order (Jacques *et al.*, 2013 ▸). This has applications in many fields of science, *e.g.* for the study of catalysts, batteries, microelectronics, micromechanical systems, nanocomposites, biomaterials, medical implants, engineering materials and corrosion.

With a degree of coherence at 80 keV similar to that currently at PETRA III at 10 keV, various phase-contrast imaging techniques become possible at significantly higher X-ray energies, in particular *in situ* and *operando* imaging in the near field and coherent X-ray diffraction imaging (CXDI) techniques, such as ptychography in the forward direction or under the Bragg condition (Godard *et al.*, 2011 ▸). Due to the flat Ewald sphere at high X-ray energies, several Bragg reflections will be accessible simultaneously, making the visualization of crystal defects possible in 3D.

## 3. PETRA IV: facility and beamline concepts   

**Figure 5:**
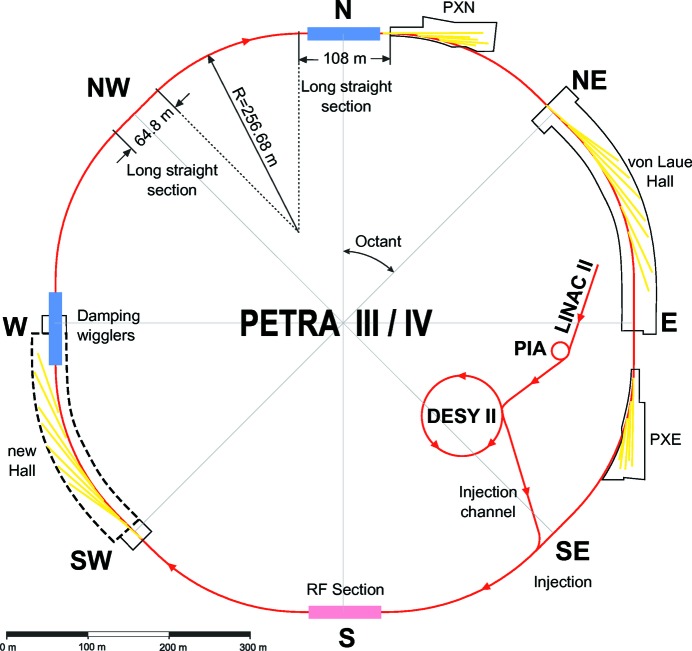
Layout of the PETRA III/IV facility.

The upgrade plan of PETRA III to PETRA IV aims at building a unique light source with an ultralow horizontal emittance in the range between 10 pm rad and 30 pm rad at an electron-beam energy of 6 GeV. The general layout of PETRA III is shown in Fig. 5 ▸. In addition to the three existing experimental halls in the northeast of the storage ring, we plan to build a new experimental hall in the southwest just opposite to the experimental hall ‘Max von Laue’.

The storage-ring design described in §4 will provide 18 insertion device sections of approximately 5 m length and four longer straight sections at the beginning of each of the four experimental halls. The current conceptual design of the storage ring seems to make so-called canting possible in a few straight sections without compromising the ultralow-emittance design goal of PETRA IV (*cf*. §4.6). In this way, two shorter undulators serving two independent beamlines can be accommodated in one insertion device section. By pursuing this canting scheme for four straight sections and splitting another four undulator beams by appropriate optics, at least 30 parallel undulator stations can be realized.

The first beamline in each experimental hall has its undulator located in one of the long straight sections separating the arcs. For these four beamlines the undulator length is not limited by the length of the insertion device section in the arc, but only by the available acceptance. For these positions the electron-beam parameters (*e.g.* β-functions) can be optimized for highest possible brightness. Fig. 4 ▸ shows the brightness that could be reached at PETRA IV with a 10 m undulator in one of the four long straight sections.

In order to fully exploit the improved brightness of PETRA IV, X-ray optics and beamline instrumentation need to be improved. X-ray optics will need to be wavefront preserving with similar requirements as for X-ray free-electron lasers (XFELs). PETRA IV can profit from the developments of these new sources; however, further developments are needed to push the numerical aperture, efficiency and quality of focusing optics to the limits. DESY pursues various such optics developments (Morgan *et al.*, 2015 ▸; Patommel *et al.*, 2017 ▸; Seiboth *et al.*, 2017 ▸). Very important is the optimization of the user operation with respect to avoiding vibrations at all relevant frequencies. This requires vibration-reduced door movements, climatization, cooling water supply, vacuum pumps, *etc.* In addition, experiments have to be especially designed to be less sensitive to external vibrations, *e.g.* by shifting resonance frequencies to high values and by active compensation. A work package on ultra-precision mechanics is part of the PETRA IV project structure.

Some experiments involving timing by pump–probe schemes or nuclear resonant scattering require sufficiently long dark times (*e.g.* ∼100 ns) between bright X-ray pulses thus requiring a reduced number of highly charged electron bunches in the storage ring. Ultralow emittance and a reduced number of electron bunches with similar total current are conflicting design goals that cannot be met with a single mode of operation of PETRA IV. Therefore, it is planned to provide at least two operation modes, a high-brightness high-coherence quasi continuous mode and a timing mode with fewer bunches with increased bunch charge but with larger emittance and thus slightly reduced brightness (*cf*. Fig. 4 ▸ and §4.4).

## 4. PETRA IV: storage ring   

Since 2009 DESY has been operating the synchrotron radiation source PETRA III (Balewski *et al.*, 2004 ▸) at a beam energy of 6 GeV and a beam emittance of 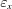 ≃ 1.3 nm rad in the horizontal and 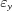 < 10 pm rad in the vertical plane. Twenty damping wigglers (total length 80 m) are used in PETRA III to reduce the emittance from 5 nm rad to 1.3 nm rad. The eight arcs (length: 201.6 m) of the PETRA ring are connected to eight long straight sections, four with a length of 108 m and four with a length of 64.8 m.

For PETRA IV an ESRF-like multi-bend achromat (MBA) lattice with a natural emittance of 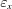 = 15 pm rad has been studied with priority. Nevertheless, the conceptual design of the lattice has not been finalized and another promising lattice option is also being considered (see §4.7). Due to the rather low *B*-field of the dipole magnets in the MBAs, the insertion devices (IDs) of the users have a strong impact on emittance and energy spread of the beam. If the IDs are installed in dispersion-free straight sections, it has been estimated that the emittance will decrease roughly by a factor of two and the energy spread will approximately double. It might become necessary to compensate gap changes of the beamline IDs by additional damping IDs (DWs) to keep the emittance and energy spread constant. In a scenario with additional damping IDs in one of the long straight sections a further reduction of the emittance to about 10 pm rad could be achieved. The basic parameters of PETRA IV (with and without DWs, and without canted beamlines) are summarized in Table 1 ▸ together with the PETRA III parameters.

### 4.1. Lattice design   

The lattice of PETRA III is based on a combination of double-bend achromat (DBA) cells with a length of 23 m in the experimental halls and FODO cells in the arc sections where no IDs are installed. In the experimental hall ‘Max von Laue’ the majority of the straight sections utilize a canting scheme with canting angles of 5 mrad and 20 mrad in the two extension halls. Our studies of an ultralow-emittance lattice have shown that canting angles in the range 2–4 mrad are acceptable without a significant increase of the emittance if undulator gaps are closed (*cf*. §4.6). As a result, most of the front-end components of the existing beamlines cannot be preserved during an upgrade to PETRA IV. This also opens up the possibility to choose a cell length larger than 23 m, which relaxes the requirements on magnet design.

For the ESRF-EBS, a special variant of the MBA, the hybrid multi-bend achromat (HMBA), was developed to mitigate the problems related to the strong sextupoles for the chromaticity correction (Biasci *et al.*, 2014 ▸; Raimondi, 2016 ▸; Wrulich *et al.*, 1992 ▸). In a seven-bend achromat, space is left between the two outer dipoles on each side to accommodate a dispersion bump. Inside the two bumps three sextupole families are installed, which are used to correct the chromaticity. The outer four dipoles have a longitudinal gradient to reduce their contribution to the emittance and to increase the height of the dispersion bump.

The dispersion bumps help to decrease the strength of the sextupoles significantly compared with the classical MBA scheme. A phase advance of 3π in the horizontal plane and π in the vertical plane between the sextupole pairs cancels most of the amplitude-dependent resonance driving terms. In the central part of the HMBA cell dipoles with horizontally defocusing quadrupole fields are used to reduce the cell length. The increase of the horizontal damping partition number due to the combined function magnets helps to decrease the emittance further.

The HMBA concept has been adopted also for the PETRA IV reference lattice. The PETRA III DBA cell is quite short (23 m) to accommodate a seven-bend achromat compared with the 26.6 m-long cell of the ESRF-EBS. Therefore, a cell length of 25.2 m and most recently of 26.2 m was used for the PETRA IV reference lattice. The beam dynamics for the longer cell are not expected to be much different from the results of the 25.2 m cell design presented here, but there is an advantage regarding magnet requirements. A cell length of 25.2 m or 26.2 m implies that the beamline configuration of PETRA III cannot be preserved. But further lattice options with a cell length of 23 m are also considered for PETRA IV (*cf*. §4.7).

**Figure 6:**
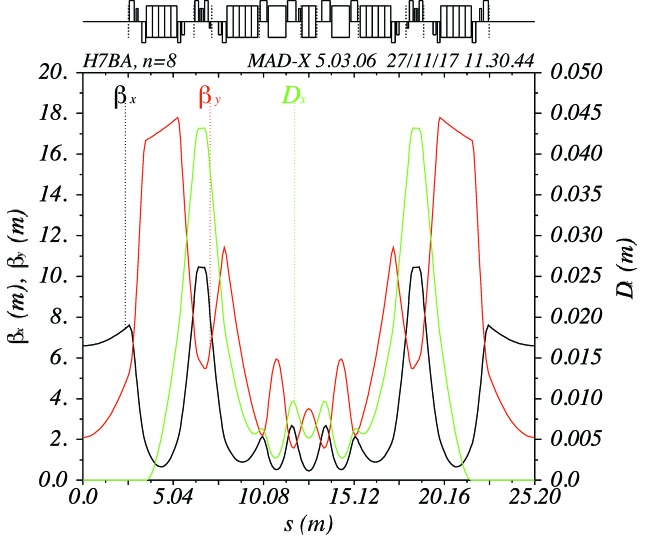
Hybrid seven-bend achromat cell of the PETRA IV reference lattice.

The hybrid seven-bend achromat cell of PETRA IV is shown in Fig. 6 ▸. The free space for the IDs has a length of about 5 m, the beta functions at the ID centre are 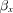 = 6.6 m and 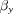 = 2.1 m. The strength of the central quadrupoles is 100 T m^−1^ for the 25.2 m cell. A longer cell length of 26.2 m helps to reduce this to ∼92 T m^−1^. The properties of a longer cell are currently under investigation.

**Figure 7:**
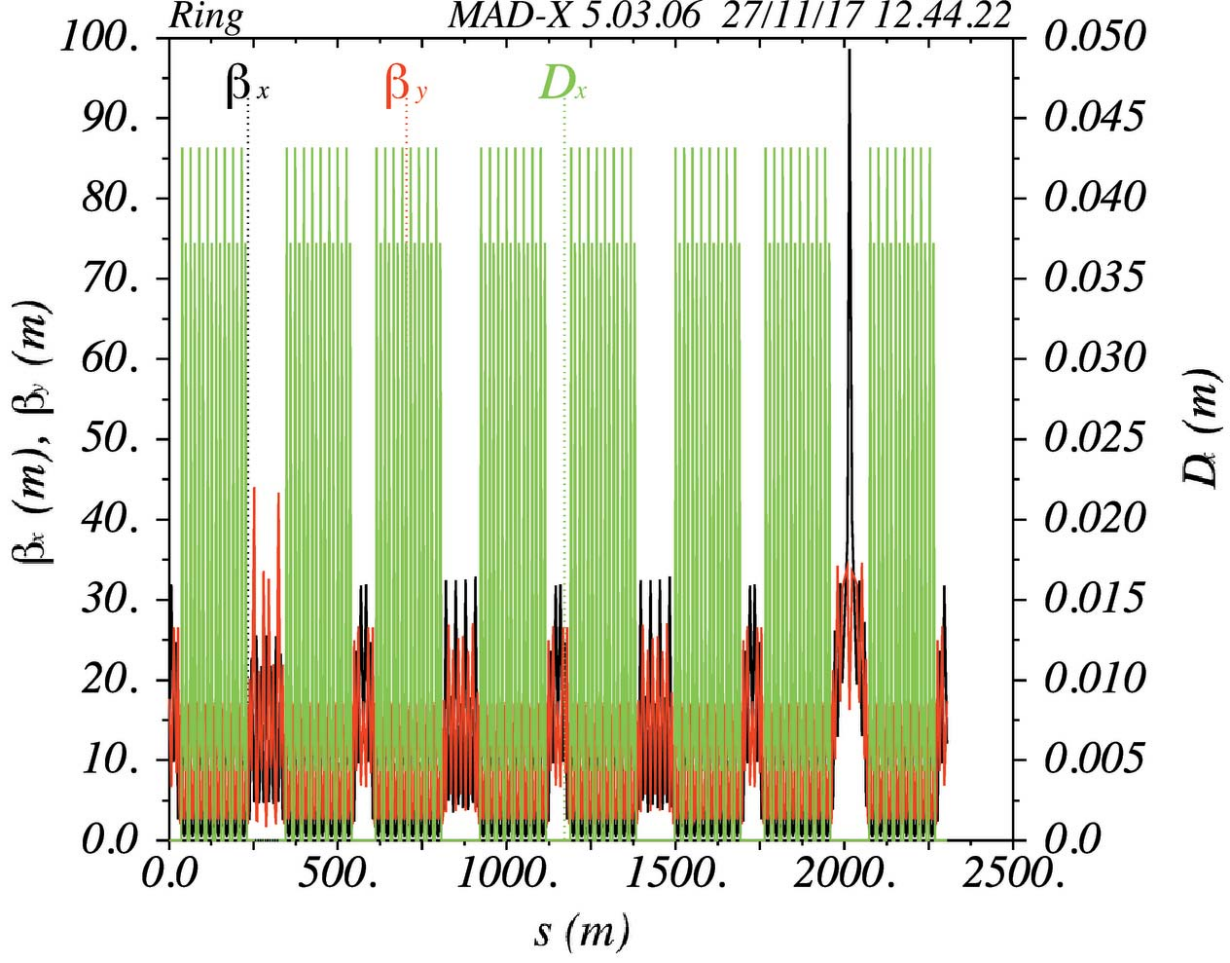
Optical functions of the reference lattice of PETRA IV.

One arc of the PETRA IV ring consists of eight identical cells of this type. The complete reference lattice has eight arcs, which are connected by eight long straight sections (Fig. 5 ▸). In one of the long straight sections an insertion with a beta function of 100 m is implemented for beam injection. In another long straight section ten damping IDs are included, each with a length of 4 m. All the long straight sections are dispersion-free (Fig. 7 ▸).

The reference lattice of PETRA IV without IDs and damping IDs provides a natural emittance of 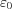 = 15 pm rad. With an additional ten damping IDs the emittance is reduced to ∼10 pm rad. As mentioned before, the IDs have a major influence on emittance, energy spread and damping times. For more realistic simulations of expected beam parameters, damping IDs have been included in the calculations.

### 4.2. Dynamic aperture and momentum acceptance   

Due to the small bending angles of the dipoles of PETRA IV the dispersion bumps are rather small compared with other machines using HMBA cells. This requires strong sextupole magnets to correct the large negative natural chromaticity. The transfer matrix of −I between corresponding sextupole pairs in the HMBA cell reduces the non-linearities induced by the sextupoles by a large amount. However, the cancellation is not perfect due to the non-interleaved sextupole scheme and the finite length of the sextupoles. To reduce them further, each arc of PETRA IV is constructed as a fourth-order geometric achromat (Cai *et al.*, 2012 ▸), making use of phase cancellation of driving terms (Verdier, 1999 ▸). By choosing a phase advance of 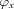 = 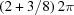 and 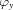 = 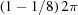 for each HMBA cell most of the third- and fourth-order geometric driving terms vanish for an arc built from eight cells. Only three amplitude-dependent tune shifts and the fourth-order resonance 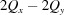 are amplified in the arcs. The driving term of the resonance can be compensated by using a phase advance difference of 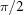 in the 64.8 m-long straight section between the planes. As the phase advance of the arcs is fixed to be a multiple of 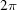, the long straight sections have to be used to set the fractional part of the tune.

The non-linear properties of the lattice can be optimized by using the harmonic sextupoles and octupoles within each cell and can also be influenced by linear optics changes within the achromat. Scanning of the linear optics parameters and the strength of multipoles of the cells (Liuzzo *et al.*, 2016*b* ▸) and multi-objective genetic algorithms (MOGA) (Borland *et al.*, 2010 ▸) have been used to enhance the dynamic aperture and momentum acceptance of the lattice.

The dynamic aperture requirement is determined by two factors: the possibility to inject the beam and the margin for beam steering required for optics measurement and calibration or photon beam alignment at the beamline. For the beam steering, we assume that a 1 mm dynamic aperture at a location with 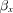 ≃ 5 m is sufficient, which translates into 0.2 mm mrad dynamic acceptance. With 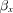 = 100 m at the injection point and assuming an emittance of approximately 10 nm rad of the incoming booster beam, the beam size is in the region of 1 mm. For on-axis injection, a dynamic aperture of 4.5 mm (or an acceptance of 0.2 mm mrad at 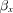 = 100 m) could already accommodate easily 4σ of the beam.

An off-axis injection is always desirable, since it allows accumulation and relaxes several requirements for the injector. For this we assume that the dynamic aperture of 10σ of the stored beam plus 4σ of the injected beam plus 3 mm for the septum blade are needed. This translates into a required dynamic aperture of approximately 7 mm or a dynamic acceptance 0.5 mm mrad.

#### 4.2.1. Dynamic aperture   

**Figure 8:**
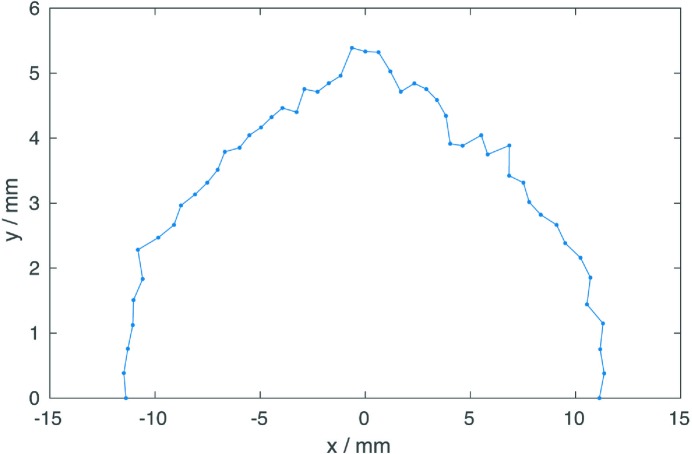
On-momentum dynamic aperture at the injection point of the reference lattice (without errors). The beta-function at the injection point is 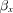 = 100 m.

To increase the dynamic aperture at the injection point the horizontal beta function in one of the long straight sections has been increased to 100 m (Fig. 7 ▸). The dynamic aperture at this point is 11 mm in the horizontal plane and 5.5 mm in the vertical plane for a lattice without errors (Fig. 8 ▸). The dynamic aperture will be significantly reduced by alignment and field errors. The sensitivity of the lattice for errors is currently studied and is discussed later on (*cf*. §4.3).

#### 4.2.2. Momentum acceptance   

**Figure 9:**
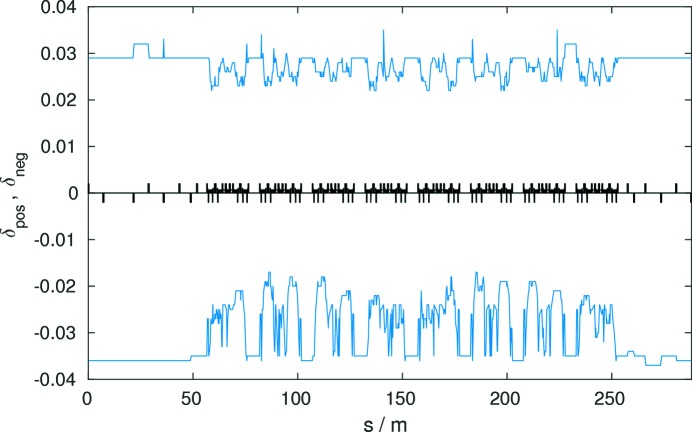
Local momentum acceptance of an octant of the reference lattice (without errors).

A sufficient local momentum acceptance is important for a large enough Touschek lifetime. The local momentum acceptance reaches ±2% in the HMBA cell sections in the regions of the dispersion bumps (Fig. 9 ▸). The momentum acceptance in the 108 m-long straight sections is around 3–3.5%. A total voltage of 6 MV of a 500 MHz RF system has been assumed.

### 4.3. Lattice sensitivity   

**Figure 10:**
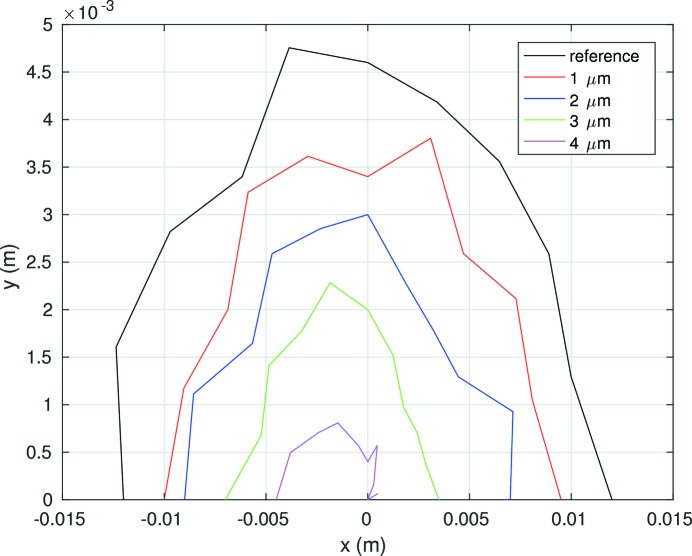
Sensitivity of the PETRA IV reference lattice to magnet misalignments.

A first estimation of the sensitivity of the dynamic aperture of the PETRA IV reference lattice to magnet misalignments is shown in Fig. 10 ▸. The dynamic aperture is evaluated by tracking 1000 turns after introducing misalignments in all magnets in both horizontal and vertical planes with a Gaussian distribution truncated at two standard deviations. Only one seed of misalignment errors is shown without any further optics or orbit correction. The dynamic aperture of the reference lattice without errors is reduced by a factor of two after introducing misalignments of 2 µm r.m.s. and it nearly disappears with misalignments of 4 µm r.m.s. The corresponding horizontal/vertical r.m.s. orbit is 136 µm/220 µm for misalignments of 4 µm r.m.s. Further studies are required to determine to what extent the dynamic aperture can be recovered after an orbit correction and which resolution for the beam position monitors is required. First estimates indicate that the PETRA IV reference lattice is more sensitive at least by a factor of two to alignment tolerances than the ESRF upgrade lattice. But it seems unlikely that an off-axis injection is still possible for the PETRA IV reference lattice if injection errors are also considered.

### 4.4. Intrabeam scattering and Touschek lifetime   

#### 4.4.1. Intrabeam scattering   

A severe limitation for low-emittance lattices is the effect of intrabeam scattering (IBS) (Piwinski, 1974 ▸; Ehrlichman *et al.*, 2013 ▸), which is a single-bunch collective effect limiting the density of the particle beam. The emittance 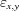 and energy spread 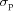 of the beam will be an equilibrium between radiation damping, quantum excitation and IBS,
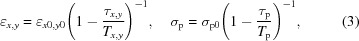
where 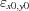 is the zero-current emittance, 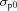 is the zero-current energy spread, 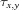, 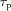 are the damping times and 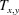, 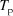 are the IBS growth rates. The additional damping due to the IDs of the photon beamlines or due to damping IDs can be used to mitigate IBS effects.

**Figure 11:**
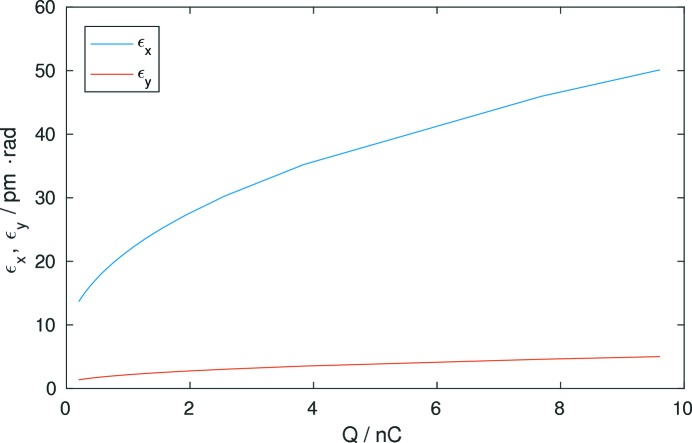
Emittance as a function of the single-bunch current due to intrabeam scattering. A coupling of 10% and a 500 MHz RF system with 6 MV has been assumed.

The equilibrium horizontal and vertical emittances due to IBS for the reference lattice are shown in Fig. 11 ▸. An emittance coupling of 10%, 40 m of damping IDs and a 500 MHz RF system with a total voltage of 6 MV has been assumed.

The emittance increase due to IBS can also be reduced by using a lower RF frequency, *e.g.* with an RF system of 100 MHz, using a higher harmonic cavity to lengthen the bunch or operating with a round beam. An additional bunch lengthening is expected by impedance effects but has not been included in the IBS calculations yet.

#### 4.4.2. Touschek lifetime   

The dominant contribution to the lifetime of a stored beam in PETRA IV is Touschek scattering of electron pairs in the transverse plane, which are then lost in the longitudinal plane (Bernardini *et al.*, 1963 ▸). Whereas in general the Touschek lifetime will decrease for smaller emittances due to the higher charge density, it will increase again for emittances <30–40 pm rad depending on the momentum acceptance. A necessary condition to obtain a sufficiently high Touschek lifetime is a large enough momentum acceptance.

The equilibrium emittances and Touschek lifetimes for a continuous mode with 960 bunches and a timing mode with 80 bunches have been calculated from the momentum acceptance of Fig. 9 ▸. The expected parameters of the reference lattice without errors are summarized in Table 2 ▸. The larger emittance in the timing mode results also in a smaller brightness in that mode. A coupling of 10% and a 500 MHz RF system with 6 MV have been assumed.

For all the IBS and Touschek calculations the simulation code *Elegant* (Borland, 2000 ▸) was used.

### 4.5. Collective effects   

**Figure 12:**
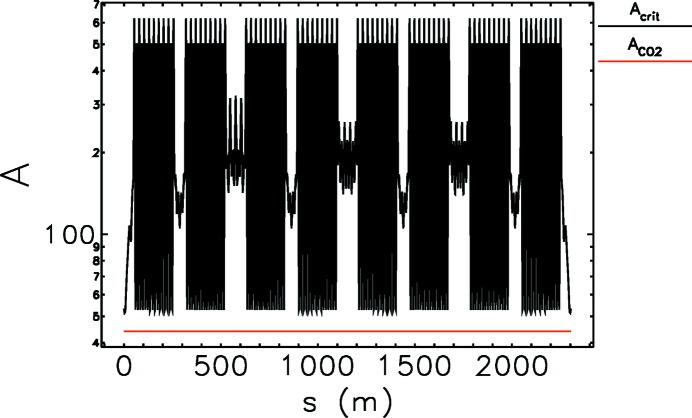
Parameter *A* for CO_2_ ions and 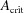 according to equation (4) shown as a function of the position along the storage ring.

The collective effects depend on the charges of the beam since a beam circulating in a storage ring interacts with its vacuum chamber surroundings *via* electromagnetic fields. These wake fields in turn act back on the beam and can lead to instabilities, which limit either the achievable current per bunch or the total current or even both. In PETRA IV we expect the space-charge effects to be small because of high energy, and effects due to electron cloud are unlikely to be observed in an electron storage ring. But, since ion instabilities were observed at PETRA III, we need to know the stability condition. Without a clearing gap, the trapping condition for an ion is expressed in a linear approximation as (Chao & Tigner, 1999 ▸) 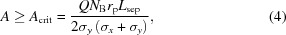
where *A* is the atomic mass, *Q* is the ion charge, *N*_B_ is the number of electrons in a bunch, *r*_p_ is the classical radius of the proton, 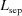 is the bunch separation and 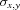 is the r.m.s. beam size. The most probable configuration for ion trapping is a multi-bunch mode filled with 960 equally spaced bunches giving a total beam current of 100 mA. For this mode we computed 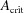 around the ring. The result for an emittance ratio 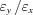 = 0.1 is shown in Fig. 12 ▸, assuming a horizontal emittance of 10 pm rad. Out of the most common gases in the vacuum chamber the heaviest CO_2_ is indicated in the graph to show the condition for no ions to be trapped according to equation (4). However, when this condition is violated for different operational modes, it will be required to introduce a clearing gap.

The wake field excited by the beam is, however, unavoidable and the small beam pipe radius will increase its effect. In the following we assume that the radius of the chamber is 10 mm and the undulator chamber’s full gap is 6 mm. A longitudinal instability was observed in PETRA III caused by long-range wake fields due to higher-order modes (HOMs) of 500 MHz cavities. In order to mitigate the unwanted beam motion a feedback system was installed and is operating regularly at 100 mA (Klute *et al.*, 2011 ▸). New cavities for PETRA IV will further improve the situation regarding HOMs. In transverse planes the dominant long-range wake is produced by the resistive wall. Due to the 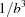 scaling its impedance will increase significantly with smaller radius *b* of the vacuum chamber compared with PETRA III. But the small-gap chambers still dominate the impedance. The contribution from the small-gap chambers is 80 MΩ m^−1^ while the contribution from the other components in the arc is only 30 MΩ m^−1^. The impedance was evaluated at the revolution frequency of 130.1 kHz assuming aluminium chambers. This is comparable with other light sources in development and the magnitude is not extreme. As a result we expect that the instability can be mitigated either by a transverse feedback system and/or by increasing machine chromaticity (Klute *et al.*, 2011 ▸; Chae, 1995 ▸).

The short-range wake field causes sudden beam losses where the feedback is too slow to counteract unstable motion. This limits the single-bunch current and its threshold can be estimated by the transverse mode-coupling instability (TMCI) for the machine at low chromaticity. The coherent frequency shift can be computed using Sacherer’s formula (Chao & Tigner, 1999 ▸). The threshold current for the TMCI is
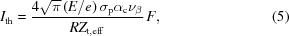
where *R* is the radius of the ring, 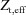 is the effective transverse impedance, 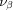 is the betatron tune, 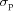 is the energy spread, 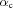 is the momentum compaction factor, and the parameter 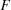 = 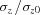 reflects the bunch lengthening by the impedance and/or by the harmonic cavity. In order to evaluate the threshold current we need to estimate the transverse impedance.

In general a small aperture increases the impedance; however, a comparative study of two chambers with different apertures (Chae *et al.*, 2007 ▸) showed that the impedances of both chambers were similar to each other. This is because the aperture-sensitive elements (BPMs) increase its impedance, while the slope-sensitive elements (undulator chambers) decrease it. Using this study as a guidance we expect that the impedance of PETRA IV will not be much different from the current one at PETRA III. Nevertheless, it is planned to extend and to refine the impedance model for PETRA IV in the future. The recent measurement showed the impedance of PETRA III to be 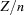 = 0.15 Ω and 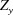 = 0.9 MΩ m^−1^ to 1.1 MΩ m^−1^ (Balewski & Wanzenberg, 2011 ▸).

Substituting the lattice parameters *E* = 6 GeV, 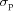 = 1.425 × 10^−3^, 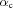 = 1.45915 × 10^−5^ and 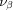 = 67.27 (vertical) into equation (5) and by using a conservative estimate on 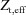 = 1.1 MΩ m^−1^, we computed the threshold current of 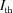 = 0.15 mA × *F*. To achieve a single-bunch current of 1 mA for the timing mode we need a bunch length of about 22 mm (r.m.s.). A preliminary study showed that a third-harmonic cavity with a moderate voltage of 1.2 MV can make such a long bunch.

The recent advance in theory predicts that increasing the head–tail phase by changes in machine chromaticity will increase the bunch current under the resitive-wall impedance (Lindberg, 2016 ▸) as well as under broadband impedance (Chin *et al.*, 2017 ▸). The findings are consistent with Sacherer’s stability criteria (Sacherer, 1974 ▸) and we have an option to operate the machine at high chromaticities (*e.g.* +5/+5) in PETRA IV if needed.

### 4.6. Canting and emittance   

**Figure 13:**
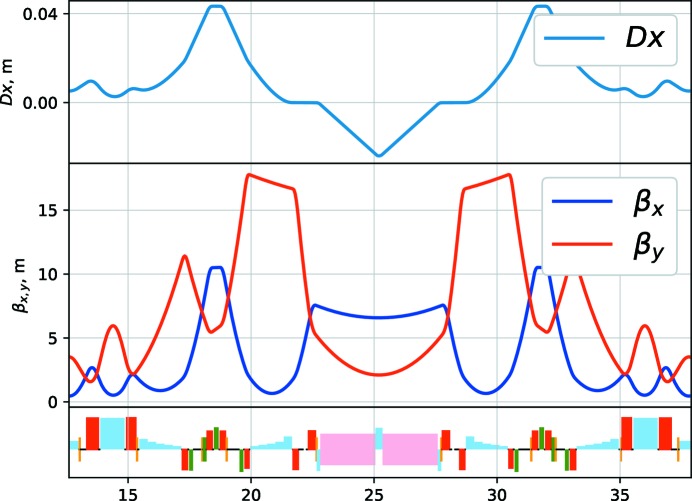
Schematic of the canting scheme with a three-magnet chicane.

**Figure 14:**
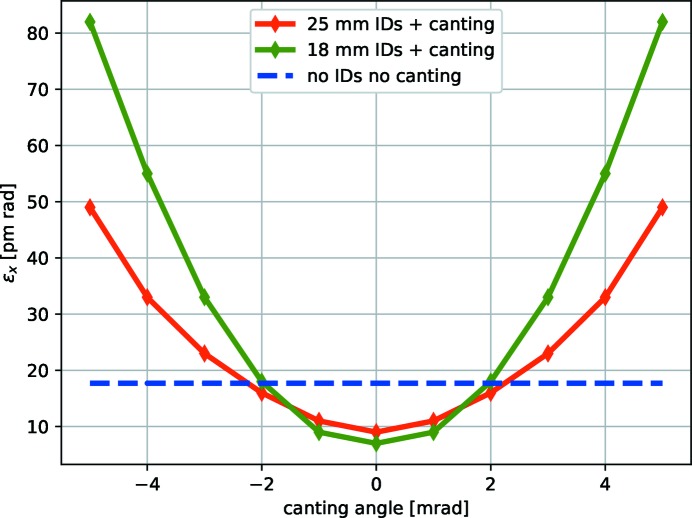
Dependency of the equilibrium emittance on the canting angle, assuming 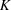 = 2, 25 mm and 18 mm devices, 5 m total length per insertion, with half of the IDs canted.

In the proposed lattice, straight sections for IDs with a length of approximately 5 m are planned. To accommodate a larger number of beamlines in the experimental halls, it is highly desirable to install two 2 m-long IDs in some of the 5 m ID sections. At PETRA III this is achieved by putting a bending magnet between the two undulators, separating the two undulator beams. For the PETRA IV lattice this scheme is still feasible; however, it disrupts the cell periodicity and thus makes adjusting non-linear characteristics of the ring non-trivial. For PETRA IV we propose a three-magnet chicane scheme, shown in Fig. 13 ▸, which does not require readjusting the optics. Undulators affect equilibrium beam emittance and energy spread, generally causing emittance damping when the dispersion is small and contributing to the energy growth. Canting results in non-zero dispersion at the ID, and the dependency of the emittance on the canting angle for two scenarios are shown in Fig. 14 ▸. Canting angles of about 2 mrad are thus feasible in the scenario, where roughly half of the straight sections use canted IDs in the three-magnet chicane scheme. Larger canting angles of, for example, 4 mrad seem to be also possible if a smaller number of insertion sections are canted. Reducing the beta function and average dispersion in the insertion section allows to further reduce the effect of canting on the emittance. More studies are needed to obtain a proper cell design to accomplish this goal.

### 4.7. Lattice options   

**Figure 15:**
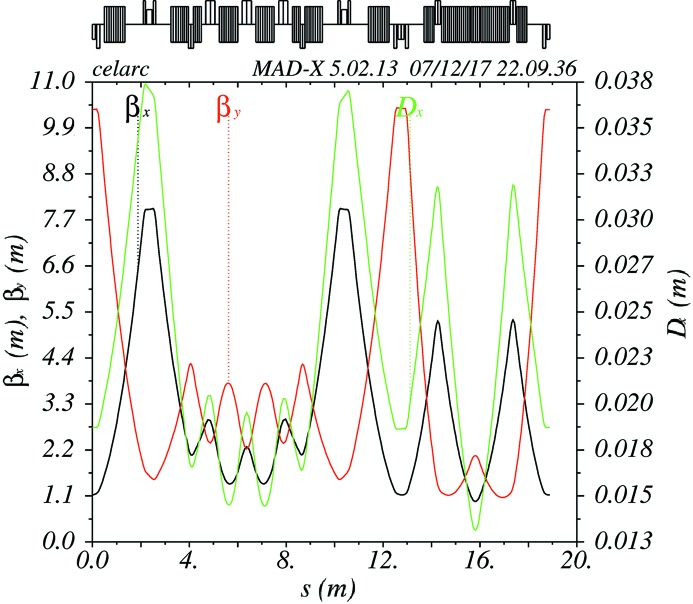
Double −I basic cell with non-interleaved sextupoles scheme with a betatron phase advance of 180° between a pair of sextupoles.

The lattice design study to convert the PETRA III storage ring into a diffraction-limited one has involved different lattice types (Agapov *et al.*, 2017 ▸). Among them, one lattice recently stands out over the rest due to the ability to potentially preserve the current off-axis injection scheme of PETRA III: the so-called ‘double −I’ lattice. Although the status of this design is still preliminary, it can serve as a proof-of-principle. This design comprises a combination of arcs based on 11 double −I cells (Fig. 15 ▸) with a non-interleaved sextupole scheme with a betatron phase advance of 180° in both planes and arcs based on eight ESRF-H7BA cells with straight sections for IDs. As is well known, such an interleaved sextupole scheme allows the effects of the geometric aberrations induced by the sextupoles to be reduced, providing a larger dynamic aperture (Brown & Servranckx, 1980 ▸). The bare horizontal emittance of such a lattice without damping IDs is about 30 pm rad. Furthermore, another cell type for the arcs with IDs of length 23 m and a FODO-type lattice will be considered in the future.

**Figure 16:**
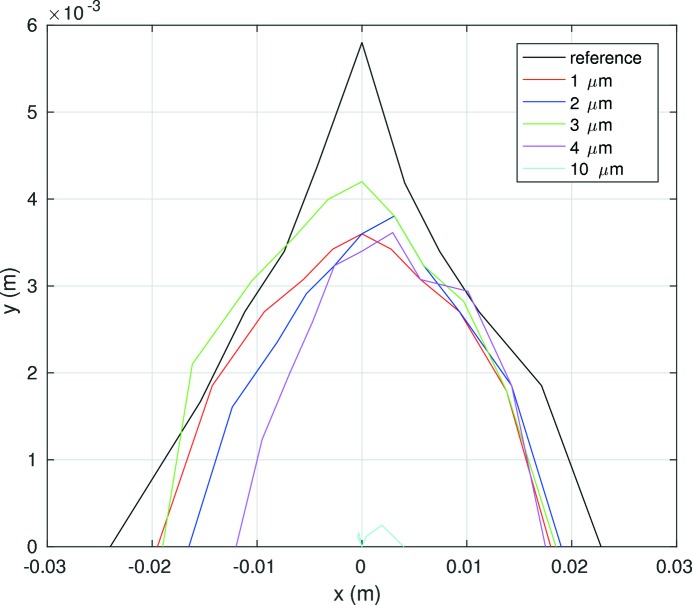
Sensitivity of the dynamic aperture of the double −I cell to different magnet misalignments.

**Figure 17:**
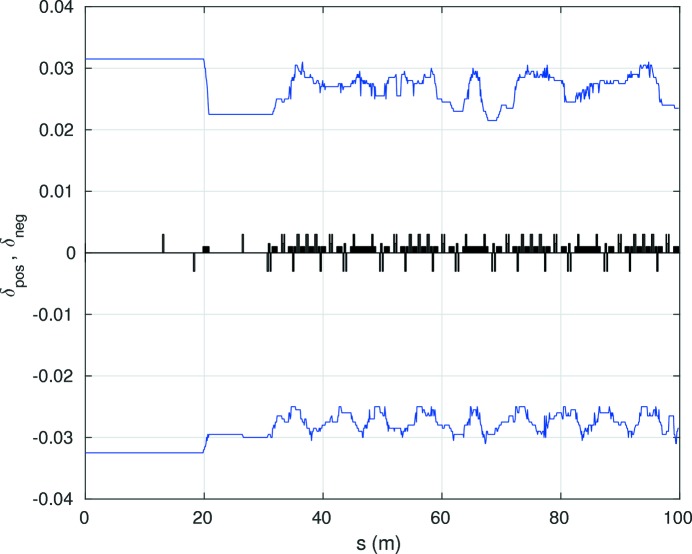
Momentum aperture of the double −I cell computed by tracking in the first part (1/16) of the ring over 1000 turns.

Indeed, the dynamic aperture without errors of the double −I lattice, shown in Fig. 16 ▸ as the black line, is larger than the dynamic aperture of the PETRA IV reference lattice shown in Fig. 8 ▸. Taking into account the fact that the betatron function in the injection point is 107.7 m, the dynamic acceptance is 4 mm mrad, three times larger than the PETRA IV reference lattice. This number exceeds the required dynamic acceptance to maintain the current off-axis injection scheme. Fig. 16 ▸ also compares the sensitivity of the dynamic aperture with magnet misalignments following the same procedure mentioned above. The dynamic aperture of the double −I lattice drops by up to 10 µm r.m.s. and hence is less sensitive to magnet errors than the reference lattice. To complement the study of the dynamic properties of this new approach, Fig. 17 ▸ shows the momentum aperture computed by tracking over 1000 turns and taking into account all elements of the first 100 m of the ring. As a result, the momentum aperture is also larger than the previous reference lattice, increasing as a consequence the beam lifetime.

### 4.8. Injection   

The injector presently used for PETRA III is the synchrotron DESY II with an emittance of 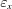 ≃ 335 nm rad and an emittance coupling ratio of 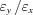 ≃ 4% (Keil *et al.*, 2017 ▸). For PETRA IV an upgrade of the injector is necessary.

**Figure 18:**
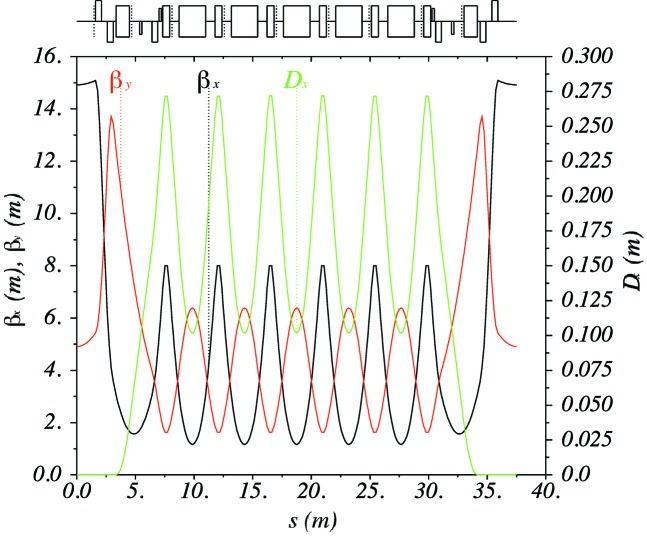
Optical functions of one octant of a new booster synchrotron.

**Figure 19:**
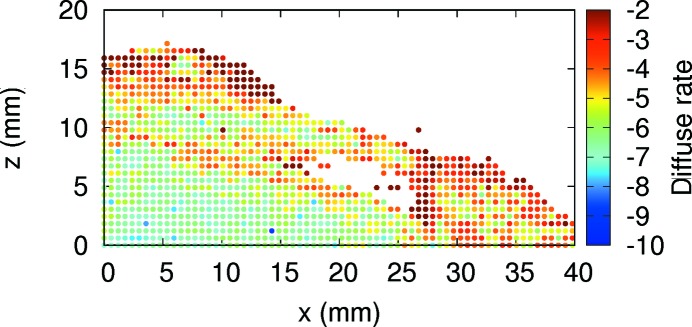
On-momentum dynamic aperture of the booster (four-dimensional tracking of 256 turns without errors).

A promising solution consisting of a modified FODO lattice with combined function magnets and achromat straights is proposed. It is obtained by scaling and adapting the booster design of ALBA (Einfeld, 2016 ▸) to a circumference of 300 m. The periodicity of the lattice is adapted to be eight, in order to fit the shape to the existing synchrotron tunnel. The equilibrium horizontal emittance at 6 GeV of this lattice is about 11 nm rad, and the 3.2 m-long straight sections are adequate for RF modules and injection/extraction septa. The Twiss functions in one octant are shown in Fig. 18 ▸ and the parameters of the booster are listed in Table 3 ▸. First tracking results (see Fig. 19 ▸) show that the on-momentum dynamic aperture of the considered lattice is larger than 20 mm, which is sufficient for an injection and extraction of the beam.

## 5. PETRA IV and the science campus in Hamburg/Bahrenfeld   

PETRA IV and the two neighbouring free-electron lasers (FELs) FLASH at DESY and European XFEL perfectly complement each other in time structure, peak power and photon-energy range. For example, PETRA IV can bridge the currently existing *temporal gap* to the FELs (*cf*. §2.2.2). At both facilities together, complex materials can be investigated over the full range of time scales from femtoseconds to minutes and hours. With a significantly lower peak power (by nine orders of magnitude) as compared with FEL sources, PETRA IV is ideal for following chemical and physical processes of an individual sample from nanoseconds to minutes and hours.

Experiments at synchrotron radiation sources and XFELs generate an increasing amount of data and require an ever-growing need for scientific computing to model physical and chemical processes and solve inverse problems associated with the measurements. These needs will be multiplied with the advent of PETRA IV and users will in most cases no longer be able to process their data without help from the facility. As part of the DESY strategy, a significant growth of the scientific computing resources and support is planned in the future to offer users the full service from remote access, data handling and storage to the complete data analysis chain.

The close proximity to various research institutions on campus, *e.g.* the European Macromolecular Biology Laboratory (EMBL), the Centre for Structural Systems Biology (CSSB), the Centre for Hybrid Nanostructures (CHyN), Centre for Free-Electron Laser Science (CFEL) and the German Engineering Materials Science Centre (GEMS) as well as the University of Hamburg, creates an ideal environment to address key questions in molecular and systems biology, nanotechnology, photon science and materials engineering. PETRA IV will have a strong leveraging effect on these institutions.

## 6. Conclusion and outlook   

In the current conceptual design phase of the PETRA IV project, the main emphasis was laid on the thorough assessment of the scientific case of an ultralow-emittance synchrotron radiation source and on developing a conceptual design for accelerators, storage ring and experimental techniques. The results will be summarized in the conceptual design report that will be compiled until the beginning of 2019. In a next step, the technical design will follow, comprising a detailed design of the accelerators and the storage ring. In addition, the beamline portfolio of PETRA IV will be developed involving the user community in a series of workshops.

## Figures and Tables

**Table 1:** Parameters of PETRA III/IV without intrabeam scattering

Parameter	PETRA III	PETRA IV (without DW)	PETRA IV (with DW)
Energy (GeV)	6	6	6
Total current (mA)	100	100†	100†
Natural emittance ∊_0_ (pm rad)	1280	15	9.3
Energy spread σ_p_ (×10^−3^)	1.23	0.73	1.44
Energy loss per turn *U*_0_ (MeV)	5.1	1.37	4.6
Momentum compaction factor α_c_ (×10^−3^)	1.13	0.0146	0.0146
Dispersion at SF *D*_*x*_ (cm)	750	4.2	4.2

† For the timing mode the total current will be 80 mA.

**Table 2:** Beam parameters in two operating modes of PETRA IV (with damping IDs), *i.e.* a high-brightness high-coherence continuous mode and a timing mode (with a 96 ns gap between bunches)

Parameter	Continuous mode	Timing mode
Bunches *B*	960	80
Bunch current *I*_b_ (mA)	0.1	1.0
Bunch charge *Q*_b_ (nC)	0.77	7.68
Horizontal emittance ∊_*x*_ (pm rad)	19.8	45.9
Vertical emittance ∊_*y*_ (pm rad)	2.0	4.6
Energy spread σ_p_	1.5 × 10^−3^	1.6 × 10^−3^
Bunch length σ_*z*_ (mm)	3.3	3.7
Touschek lifetime τ_t_ (h)	3.9	0.5

**Table 3:** Booster parameters

Periodicity	8
Circumference	300 m
Harmonic number	500
Straight length	3.2 m
Working tune	(17.17, 12.38)
Natural chromaticity	(−26.95, −15.80)
Horizontal damping partition	2.25
Momentum compaction	2.17 × 10^−3^
Energy loss	6.67 MeV
Bending field	1.16 T
Equilibrium emittance	10.7 nm rad
Equilibrium energy spread	2.03 × 10^−3^
Horizontal damping time	0.80 ms
Vertical damping time	1.80 ms
Longitudinal damping time	2.44 ms
